# Implementation of motivational interviewing in a fall prevention exercise program: experiences from a randomized controlled trial

**DOI:** 10.1186/s13104-019-4309-x

**Published:** 2019-05-14

**Authors:** Marina Arkkukangas, Staffan Hultgren

**Affiliations:** 1 Department of Neuroscience, Physiotherapy, Uppsala University, Box 593, 751 24 Uppsala, Sweden; 2 Center for Clinical Research Sörmland, Uppsala University, Uppsala, Sweden; 3 Region Västmanland, 721 89 Västerås, Sweden

**Keywords:** Older adults, Exercise, Falls, Physiotherapist, Behavior

## Abstract

**Objective:**

The elderly population over 65 is increasing globally, and interventions promoting health and preventive work, especially fall prevention, will constitute a large part of physiotherapists’ duties in the near future. To address the challenges of promoting effective and sustainable health behavior changes among older persons, physiotherapists need support when it comes to how to apply behavior change strategies, especially in fall prevention. Therefore, the aim of this study was to describe implementation of motivational interviewing in a fall prevention exercise program. This study is a side product of another project.

**Results:**

Data from a recently performed three-armed randomized controlled trial were used to describe the implementation of motivational interviewing in the exercise group (n = 58). Level of motivation (priorities) and self-efficacy for both the physiotherapist and the participant in treatment, and to use a guide targeted towards the planned treatment are recommended actions. Regular meetings and follow ups as well as updates of motivational interviewing skills during a treatment period, should also be considered to achieve treatment fidelity.

*Trial registration* NCT01778972, Retrospectively registered January 29, 2013

## Introduction

The older population over 65 is increasing globally and is estimated to nearly triple in the next 30 years to 1.5 billion [1]. At the age of 65, approximately 35% of all older adults experience falls [1] and strategies for promoting health behavior changes that target the older population are needed in the field of physiotherapy, especially when promoting fall prevention [2, 3]. However, older adults’ engagement in physical activity and exercise is generally low, confirmed in a study by Basset [4] discussing the problematic issue with non-compliance to physiotherapy prescriptions and non-attendance to physiotherapy sessions and being non-adherent to home-based exercise programs [4].

Behavior change strategies in physiotherapy have been evaluated to some extent in studies of middle-aged people and in the research area of pain management among older, frail women. Recommended behavior change strategies are requested in other areas such as in fall prevention in physiotherapy [5, 6]. Two factors have been found to be important in interventions that promote physical activity among older people: motivation and self-efficacy. [7, 8] One communication method commonly used to elicit behavior change is motivational interviewing (MI) [9]. MI was developed in 1980–1990 by psychologists William Miller and Stephen Rollnick. MI was developed to help people discover and address ambivalence regarding behavioral change [10]. MI is well known as an effective method for addressing abuse/addiction, but it has also been applied in modern health care when promoting health behavior changes, such as physical activity, obesity, hypertension, and diabetes [11–13].

The use of MI in the field of fall prevention in physiotherapy is sparsely investigated. To our knowledge the RCT study by Arkkukangas et al. [14] is the first study using MI in combination with an evidence-based fall prevention exercise program in a three-armed RCT, with one of the arms (n = 58) using MI combined with fall preventive exercise. Therefore, the aim was to describe implementation of MI in a 1-year fall prevention exercise program among community-dwelling older adults in Sweden.

## Main text

### Procedure

This study used a descriptive investigation of a previous performed RCT. The study interest was implementation of MI in the intervention group receiving fall prevention exercise supported by MI in the RCT study. Five studies have previously been published based on the RCT presenting; feasibility, effects in short and long term follow up, experiences, and adherence to exercise [14–18].

### Study intervention

The RCT consisted of a 1-year intervention with follow up visits by a physiotherapist (PT) five times during the first 3 months and with one additional visit at 9 months, a total of six visits. In addition, three follow-up phone calls were made during 1 year of exercising. The participants were community-dwelling adults over 75 years of age who were able to walk independently with or without walking aids. Cognitive ability was measured by the Mini Mental State Examination (MMSE), and the set of clients who scored over 24 (with a maximum score of 30) were included in the study. The fall-preventive exercise was based on the home-based Otago Exercise Programme (OEP), an individualized exercise program developed in New Zealand by Campbell et al. [19]. Implementation of the OEP has been described by Gardner et al. [20]. The addition of MI was used to enhance the participant’s own motivation for exercise behavior change, using open-ended questions, affirmations, reflective listening and summaries (OARS) [9]. Throughout all the sessions underlying principles of MI were present (Table 1).

**Table 1:** The MI guide developed for the exercise intervention

MI concepts	Procedure
1. Engaging, focusing and evoking	Create relationship *I appreciate our meeting today* *Where do you want us to sit down and talk?* Agenda: At this meeting we will talk about why I’m here, and above all, I’m interested in your thoughts and experiences of exercising. I will later perform some tests and exercises, leading to an exercise program for you *How does this sound to you?* Use OARS—Open-ended questions, affirmation, reflective listening and summarize
*EX: Strategies for information*	*Ask permission to give information about the exercise* *Information: we will meet five times during a 3*-*month period and after that will have slightly sparse contact for up to 1* *year*
*Strategies to go back to the agenda*	*You have so many interesting things to share with me. I truly appreciate you wanting to share this with me, and we can return to this*—*link back to the agenda and why you are here*
2. Focus on change talk	*What are your exercise experiences?* To recognize, elicit and respond to change talk
*DARN*	*Desire (I would like to, I wish…)* *Ability (I think I could…)* *Reasons (I know I would feel better…)* *Need (I should exercise at least…)*
*CATs*	*Commitment (intention, decision…)* *Activation (prepared to…)* *Taking steps (doing…)*
3. Response to change talk—EARS	*Elaborating (In what way, examples…)* *Affirmation (Positive comments of statements)* *Reflecting (Can never reflect too much!)* *Summarizing (Collect bouquets of change talk)*
*Tests and introducing exercises*	*Perform the tests and the individualized adjusted exercises* *Now, I have some questions that I would like to ask you to rate on a scale from 1*-*10 (Importance, ability, confidence and satisfaction)*
*Rating scales*	*Examples of follow up questions* *Why do you rate…and not zero?* *At a low rate 5*-*6, how is it/why don´t you rate less?* *At a low rate, exercise does not seem to be important to you currently!* *At a low rate, what would you need to rate higher?* *What is your (strength, ability) that would help you accomplish this?* *If you decide to follow this program, how would you succeed?* *If you decide to do this, you will complete it!*
4. Planning	Planning occurs when commitment is achieved AND there is a clear goal for change AND there is clear motivation on the part of the person who is attempting change. Use SMART-specific, measurable, achievable or attainable, realistic and timed
5. Maintenance	How would you go about performing the exercise?
6. Final	What do you think of all this?

### Intervention outcomes

In the RCT, the participants performing exercise supported by MI improved their physical performance (p = .04), fall self-efficacy (p = .02), activity level (p = .02), and handgrip strength (p = .03) after 3 months of exercising [15]. However, at the 1 year follow up assessment, no significant improvements could be detected for the group even though such improvements had been noted at 3 months [16]. The PTs performing the MI intervention were highly motivated and expressed that they were competent in using MI when introducing fall-preventive exercise for older persons [14]. Goal setting for the exercise program were also highlighted. The older participants expressed that having a pronounced goal was an important factor in performing the prescribed exercise [17]. One of the most beneficial effects from the exercise period was that performing exercise with the addition of MI was positively associated with adherence to the exercise regimen at the 1 year follow up when exercising twice a week [18].

### MI education and support

The PTs introduced fall-preventive exercise, with the addition of MI, in the participant’s home. All the PTs were familiar with, and educated in, MI and had undergone a 3-day training program with a Motivational Interviewing Network of Trainers trainer, (MINT trainer) prior to the start of the study. In collaboration with the MINT trainer an MI guide was developed during the training program. The purpose was to support the PTs in the collaborative conversation with the older adults. Results from a questionnaire about the MI guide revealed that it was more of a hindrance than a support for the PTs [14]; and more support from the MINT trainer was requested. In addition, selected recorded conversations were coded at the Motivational Interviewing Coding Laboratory (MIC lab) in Sweden. The results of the coding scored an average of 2.4 rated on a 5-point Likert scale, where 0 indicated little to no mastery over MI techniques and 5 indicated an expert level of mastery over MI techniques. This indicated the need for more support. Two booster sessions were performed after the results from the first coding were obtained. The physiotherapists expressed that, when engaging in more conversations, their skills seemed to have improved, and they were more confident and secure in their performances. A second coding was made at the MIC lab, and an acceptable average score of 3.8 was achieved [15]. In the additional meetings with the MINT trainer, a new MI guide was constructed. It was offered as a support tool to be used before and between meetings with the participants to remind physiotherapists of MI techniques and to keep their MI skills updated during the rest of the intervention period.

### Identification of motivation and self-efficacy

As evidenced by prior experience in introducing and prescribing exercise for older persons, one of the most common situations to arise involves a lack of investigation in a client’s motivation to change [9, 10]. This initial consultation with a patient in physiotherapy is critical for understanding what possible engagement the PT can expect from the older adult [12]. The older participants in the RCT rated their motivation, placing an emphasis on the priorities surrounding their exercise and on their self-efficacy in performing the exercise, both in the beginning of the exercise period and at the 3 month and 9 months follow ups in the RCT. Statistically significant difference were detected for the ability to perform exercise (p = .03) at 3 months, compared to baseline. The satisfaction (p = .004) of performing exercise at the 3 months follow up also showed statistically significant difference compared to at the 9 months follow up, therefore support should be provided for a longer period than 3 months.

Another overlooked aspect involves the motivation expressed by both sides, both by the PT and the person making a change. From the PT’s perspective, the motivation to be the person with skills to support the client in making a behavior change is perhaps the most important assessment to consider; this assessment should include both the PT’s own motivation and competence in the situation. In the feasibility study of the RCT [14], the motivation levels of PTs were rated as high, on a scale from 0 to 10, with a median rating of 10 [14]. In addition, their competence levels were rated as very high (yes/no) (82%). These ratings indicate the PTs level of engagement with the treatment and the need for proper education and support.

### Using a guide

The PTs in the RCT used a guide designed especially for the study to prepare for the collaborative conversations between the PTs and the participants, see Table 1. All concepts were briefly explained in the guide, and some examples were provided: (Table 1). The meetings were set in the participant’s home; the PTs started the sessions with a collaborative conversation during the first visit, and after this first visit, exercises were integrated with MI during the rest of the sessions.

### Clinical recommended aspects

Recommended aspects to include in planned fall-preventive exercise treatment based on experiences from the RCT; (1) Use a guide targeted towards the planned treatment. (2) Level of motivation (priorities) and self-efficacy for both the physiotherapist and the participant in treatment. (3) Regular meetings and updates of MI skills (at least every 6 months). (4) Regular follow ups during the whole treatment period (telephone, technology-based, or physical meeting).

### Discussion

The aim of this study was to describe the implementation of MI in a fall prevention exercise program and add to the practical implementation of MI in physiotherapy. This study highlights the importance of regular follow ups and regular support during a treatment intervention. Further MI skills follow ups and coding of MI skills should be performed regularly to ensure treatment fidelity [21]. This finding is one of the most important results of our study, since MI education for health care professionals, especially in Sweden, is performed only once, sometimes with an opportunity to add an advanced MI education. Health care professionals and their employers should be aware that MI skills need to be updated and used regularly for the essence and skills of MI to stay intact. If these actions are ignored active ingredients of MI may be lost [22]. In addition, identification of motivation is important to investigate, both from the physiotherapist’s and the participant’s perspective (planned and performed exercise). The ability to perform the prescribed exercise was significantly higher at 3 months compared to the baseline, and the satisfaction with the prescribed exercise was significantly higher at 3 months compared to 9 months, suggesting that the support provided during the first 3 months is important. This finding also correlates with previous research into supervised vs. unsupervised exercise, which found home based exercise to be the least effective compared to group exercise or exercise supervised at a health care center [23, 24].

Fall preventive exercise supported by MI positively predicted exercise adherence when exercising twice a week during 1 year in the RCT [18], suggesting that support provided during the intervention contributed to an exercise behavior change for this study sample, despite the indication of more support needed after 3 months participation in the study. Nevertheless, the amount of support and the most beneficial lengths and types of behavioral interventions are yet to be determined, since, in the RCT, some effects detected at the three-month follow up were no longer present at the 1-year follow up [16]. Based on the results from this study, it is recommended that continued research in this area of physiotherapy be conducted and prioritized, targeting the amount of support and the most beneficial lengths needed for fall prevention exercise behavior change.

### Conclusion

Physiotherapists could benefit from using MI in a structured way in fall prevention when striving for fall prevention exercise behavior change. Implementation of MI in a fall prevention program should include using an MI guide and regular support to maintain MI skills. Investigation of motivation from both physiotherapist and the person in treatment should be of high importance to achieve long term fall preventive exercise adherence.

## Limitations

Since this study is a side product of another project, MI implementation were not the primary target in the main project. However, the complexity in using behavior change strategies in interventions needs more attention, therefore we judge this study to be clinically important. Also, a conclusion of what possible effects MI might have had in this context should be handled with caution, since the main outcome effects from the exercise were absent at 1-year follow up. Since exercise adherence is a major challenge, we still judge this study to be of interest and an indication to be further investigated in future research.

## Data Availability

Data sharing is not applicable to this article as no datasets were generated or analyzed during the current study.

## Abbreviations

OEP: Otago Exercise Programme
MI: motivational interviewing
MINT: motivational interviewing network of trainers
MMSE: mini mental state examination
RCT: randomized controlled trial
PT: physiotherapist
